# Understanding Altered Dynamics in Cocaine Use Disorder Through State Transitions Mediated by Artificial Perturbations

**DOI:** 10.3390/brainsci15030263

**Published:** 2025-02-28

**Authors:** Yi Zheng, Yaqian Yang, Yi Zhen, Xin Wang, Longzhao Liu, Hongwei Zheng, Shaoting Tang

**Affiliations:** 1 School of Mathematical Sciences, Beihang University, Beijing 100191, China; 2 Key Laboratory of Mathematics, Informatics and Behavioral Semantics, Beihang University, Beijing 100191, China; 3 Institute of Artificial Intelligence, Beihang University, Beijing 100191, China; 4 Zhongguancun Laboratory, Beijing 100094, China; 5 Beijing Advanced Innovation Center for Future Blockchain and Privacy Computing, Beihang University, Beijing 100191, China; 6 State Key Laboratory of Complex & Critical Software Environment, Beihang University, Beijing 100191, China; 7 Beijing Academy of Blockchain and Edge Computing, Beijing 100085, China; 8 Hangzhou International Innovation Institute, Beihang University, Hangzhou 311115, China; 9 Institute of Medical Artificial Intelligence, Binzhou Medical University, Yantai 264003, China

**Keywords:** cocaine use disorder, brain states, resting-state functional MRI, whole-brain modeling, perturbation

## Abstract

**Background/Objectives**: Cocaine use disorder (CUD) poses a worldwide health challenge, with severe consequences for brain function. However, the phase dynamics underlying CUD and the transitions between CUD and health remain poorly understood. **Methods**: Here, we used resting-state functional magnetic resonance imaging (fMRI) data from 43 CUD patients and 45 healthy controls (HCT). We performed empirical analysis to identify phase-coherence states and compared their probabilities of occurrence between conditions. To further explore the underlying mechanism, we employed computational modeling to replicate the observed state probabilities for each condition. These generated whole-brain models enabled us to simulate external perturbations and identify optimal brain regions mediating transitions between HCT and CUD. **Results**: We found that CUD was associated with a reduced occurrence probability of the state dominated by the default mode network (DMN). Perturbing the nucleus accumbens, thalamus, and specific regions within the default mode, limbic and frontoparietal networks drives transitions from HCT to CUD, while perturbing the hippocampus and specific regions within the visual, dorsal attention, and DMN facilitates a return from CUD to HCT. **Conclusions**: This study revealed altered DMN-related dynamics in CUD from the phase perspective and provides potential regions critical for state transitions. The results contribute to understanding the pathogenesis of CUD and the development of therapeutic stimulation strategies.

## 1. Introduction

With over 20 million individuals worldwide misusing cocaine, cocaine use disorder (CUD) has emerged as a significant global health problem [1,2]. CUD is primarily characterized by cocaine cravings, compulsive drug-seeking, and impaired control over use [3,4]. CUD leads to severe consequences on cognitive function, including impaired emotional regulation, attention deficits, and diminished response inhibition [5,6,7,8]. Additionally, CUD shows high relapse rates and lacks effective treatments. Therefore, uncovering the underlying dynamics of CUD and exploring potential therapeutic approaches have become key focuses in neuroscience and psychiatry.

Functional magnetic resonance imaging (fMRI)-based studies have advanced our understanding of the neural mechanisms underlying CUD by exploring functional connectivity (FC) differences. CUD is associated with widespread functional alterations in the brain [9]. For instance, FC changes between the striatum and prefrontal regions impact compulsive drug use and trait impulsivity [10]. Furthermore, significant FC alterations in the default mode network (DMN) have been associated with impaired self-awareness, negative emotions, and addiction-related rumination [11]. However, prior research has mainly focused on static FC, overlooking temporal fluctuations in connectivity [12,13]. Dynamic functional connectivity analysis overcomes this limitation and offers richer insights into the brain’s dynamic states. Time-varying information can be extracted using the Leading Eigenvector Dynamics Analysis (LEiDA) by computing the inter-regional phase coherence across the scan [14]. This method offers several advantages compared to other dynamic functional connectivity methods (e.g., sliding window analysis), including dimensionality reduction by focusing on the leading eigenvector, which simplifies the data and enhances computational efficiency; robustness to noise, as the leading eigenvector is less susceptible to high-frequency noise; and an enhanced signal-to-noise ratio, which improves the detection of meaningful connectivity patterns [14]. It further identifies distinct metastable states with the corresponding occurrence probabilities, creating a probabilistic metastable substate (PMS) space that can characterize different conditions [15,16,17]. The LEiDA method has been successfully applied to studies of cognitive tasks [18], aging [14,19], sleep [20], and different psychiatric conditions [21,22], yet its application to CUD remains limited [23,24,25]. In particular, little is known about the temporal dynamics of inter-regional synchronization from the phase perspective in CUD.

Artificial stimulation techniques have been widely used to explore brain mechanisms and their causal relationships with behaviors [26]. Stimulation approaches enable direct modulation of neural activity in targeted areas with the effects spreading throughout the whole brain, offering promising therapeutic avenues for neurological and psychiatric disorders. Deep brain stimulation (DBS) is frequently used for treating Parkinson’s disease, Alzheimer’s disease, and dementia [27,28,29], while non-invasive transcranial magnetic stimulation (TMS) is commonly employed for treating epilepsy, autism, and schizophrenia [30,31,32]. Notably, TMS has also shown potential for mitigating addiction-related symptoms in CUD [33]. However, the influence of local brain regions on whole-brain dynamics and the identification of optimal brain regions for effective CUD stimulation treatment remain unclear [34,35,36].

The dorsolateral prefrontal cortex (DLPFC) is commonly considered a key target for TMS treatment of CUD. Recent evidence suggests that regions within the frontoparietal control and default mode networks may serve as additional potential targets for therapeutic intervention [37,38]. Experimentally examining the stimulation effects on different brain regions is impractical, time-consuming, and potentially harmful. Computational modeling provides a powerful approach to investigating these unknown situations [39,40]. Computational modeling in addiction research is broadly categorized into three primary frameworks: reinforcement learning models, Bayesian and active inference models, and neural models [41,42]. The first two approaches simulate addictive behaviors and cognition through mechanisms including reward prediction errors and maladaptive belief updating [43,44]. In contrast, neural models focus on alterations in dopamine-mediated cortico-striatal circuits and local brain dynamics [45,46]. However, the application of whole-brain dynamical modeling to investigate the neural activities associated with addiction is still lacking. In this work, we utilized the whole-brain Hopf model originally proposed by Deco et al. [20]. This framework provides mechanistic insights into brain-state organization—specifically, it elucidates how large-scale neural interactions emerge from anatomical coupling mechanisms. Crucially, the model enables artificial stimulation simulations through targeted modifications of local bifurcation parameters. Such a perturbation strategy allows systematic investigation of how region-specific activity alterations propagate through the network, ultimately reshaping brain-state dynamics and driving transitions between pathological and healthy conditions. This bifurcation-based perturbation framework has been applied to promote transitions from aging [47], major depressive disorder [48], schizophrenia [49], and unconscious states [50] toward healthier conditions.

Here, we aimed to investigate the abnormal dynamics of CUD and potential transitions between pathological and healthy conditions. Based on evidence of abnormalities in higher-order cognitive regions [10,25], particularly within the DMN [11], and the critical role of metastable dynamics in psychiatric disorders [21,22,48,49], we hypothesize that compared to healthy controls (HCT), CUD patients would exhibit altered probabilities of DMN-associated state probabilities; and perturbations in higher-order cognitive regions would modulate brain-state transitions, steering the system toward either the pathological or healthy condition. To test the hypotheses, we first characterized and compared the dynamic states of HCT and CUD groups using LEiDA. Next, we constructed a whole-brain model and replicated empirical state probabilities through model fitting and optimization. The generated effective connectivity incorporated both structural and functional information, reflecting the differences in causal mechanisms between different groups. Finally, we simulated the effects of local perturbations on the HCT and CUD models and identified the brain regions most effective at facilitating transitions between groups.

## 2. Materials and Methods

### 2.1. Participants and MRI Data Acquisition

The main analysis used data from the publicly available SUDMEX-CUD neuroimaging dataset (Mexico) [51]. This dataset adhered to the principles of the Declaration of Helsinki and received approval from the Ethics Committee of the Instituto Nacional de Psiquiatría, Mexico. All participants provided informed consent. After rigorous screening of MRI and demographic data, the study included 88 individuals (84% male, mean age = 30.90 ± 7.86, age range = 18–50), comprising 45 healthy controls and 43 cocaine use disorder patients.

Briefly, the resting-state fMRI data were collected using a gradient-recalled echo planar imaging sequence with the following parameters: dummies = 5, TR = 2000 ms, TE = 30.001 ms, 3.0 mm isotropic voxels, flip angle = 75°, field of view (FOV) = 240 × 240 mm, slice acquisition order = interleaved (ascending), slice number = 36, and phase encoding direction = AP. Each scan lasted 10 min. Participants were requested to keep their eyes open and fixate on a cross during scanning. T1w data were collected using a 3D FFE SENSE sequence with TR = 7 ms, TE = 3.5 ms, field of view (FOV) = 240 × 240 mm, slice number = 180, plane = sagittal, 1.0 mm isotropic voxels (first five participants were collected with a voxel size = 0.75 × 0.75 × 1 mm). DWI-HARDI data were collected using a SE sequence with TR = 8600 ms, TE = 126.78 ms, field of view (FOV) = 224 × 224 mm, slice number = 50, plane = axial, 2.0 mm isotropic voxels, directions: 8 = b0, 36 = b-value 1000 s/mm^2^ and 92 = b-value 3000 s/mm^2^, total = 136 directions. Further details on participant selection and MRI acquisition protocols are provided in [51].

This study also used functional data from the SUDMEX-TMS neuroimaging dataset (Mexico) for robust validation of state dynamics [52]. This dataset adhered to the principles of the Declaration of Helsinki and received approval from the Ethics Committee of the Instituto Nacional de Psiquiatría, Mexico. All participants provided informed consent. After excluding data with large head motion, the analysis included 48 CUD subjects (85% male, mean age = 35.27 ± 7.52, age range = 18–48). The resting-state fMRI data were collected using a gradient-recalled echo planar imaging sequence with the following parameters: dummies = 5, TR = 2000 ms, TE = 30.001 ms, voxel size = 3 × 3 × 3.33 mm, flip angle = 75°, field of view (FOV) = 240 × 240 mm, slice acquisition order = interleaved (ascending), slice number = 36, and phase encoding direction = AP. Each scan lasted 10 min. Participants were requested to keep their eyes open. T1w data were collected using a 3D FFE SENSE sequence with TR = 7 ms, TE = 3.5 ms, field of view (FOV) = 240 × 240 mm, slice number = 180, plane = sagittal, 1.0 mm isotropic voxels (five participants were collected with a voxel size = 0.75 × 0.75 × 1 mm). Further details about the MRI acquisition protocols are provided in [52].

### 2.2. Diffusion and Functional MRI Pre-Processing

We applied quality control procedures on the SUDMEX-CUD dataset to minimize nuisance noise. The exclusion criteria were as follows: (1) DWI images showing outliers (1.5 times the interquartile range in the forward or adverse direction) in DSI Studio-derived quality metrics, including DWI contrast neighboring, DWI correlation neighboring, DWI correlation (masked), and bad slices; (2) functional scans with more than 7% of time points classified as motion spikes (framewise displacement ≥ 0.5 mm), average framewise displacement exceeding 0.4 mm, or presence of any motion spike larger than 5 mm; (3) visual quality inspection; and (4) cases with missing MRI images or incomplete demographic information (group, gender, and age). After applying these criteria, the final sample consisted of 88 participants.

The replication dataset included longitudinal data from 54 CUD patients undergoing treatment, of which only baseline (pre-treatment) functional data were used. Given the relatively small sample size, we adopted less stringent motion criteria, excluding functional scans if the average framewise displacement exceeded 0.5 mm or if any motion spike larger than 5 mm was present. This resulted in 48 CUD patients, comparable to the number of HCT subjects (n = 45) in the main analysis.

Functional data preprocessing was conducted using fMRIPrep (Stanford, CA, USA) [53,54]. The software performed intensity non-uniformity correction, skull stripping, segmentation, nonlinear registration, and volumetric normalization into two standard spaces for each T1w image. Each BOLD sequence underwent head motion correction, slice timing correction, and co-registration to the T1w reference. Confound time series were calculated, including framewise displacement, CompCor components, and motion parameters. The preprocessed BOLD data were further denoised using XCP-D (Philadelphia, PA, USA) [55]. This procedure included removal of dummy volumes, regression of top 5 aCompCor components, six motion parameters and their derivatives, despiking, cubic spline interpolation for high-motion outliers, band-pass filtering (0.01–0.08 Hz), and Gaussian spatial smoothing (FWHM = 6 mm). The denoised data were parcellated into regional time series by computing the mean voxel-wise activity within each node of the 116-node atlas [56,57], consistent with established practices in prior neuroimaging studies [20,50].

Diffusion data preprocessing and structural connectivity (SC) construction were performed using DSI Studio (Pittsburgh, PA, USA). Briefly, the QSDR algorithm was applied for MNI space registration (Pittsburgh, PA, USA) [58]. Deterministic tractography (DSI Studio’s modified FACT algorithm, Pittsburgh, PA, USA) was used to generate 1,000,000 streamlines with parameters consistent with previous research: angular cutoff = 55°, step size = 1.0 mm, minimum streamline length = 10 mm, maximum length = 400 mm, spin density function smoothing = 0.0 [59,60,61]. SC between region i and region j was defined as the mean of the proportion of fibers from region i reaching region j and that from region j to region i [49]. Group-level SC matrices were derived by averaging individual matrices within the HCT and CUD groups.

### 2.3. Leading Eigenvector Dynamics Analysis

**Figure 1:**
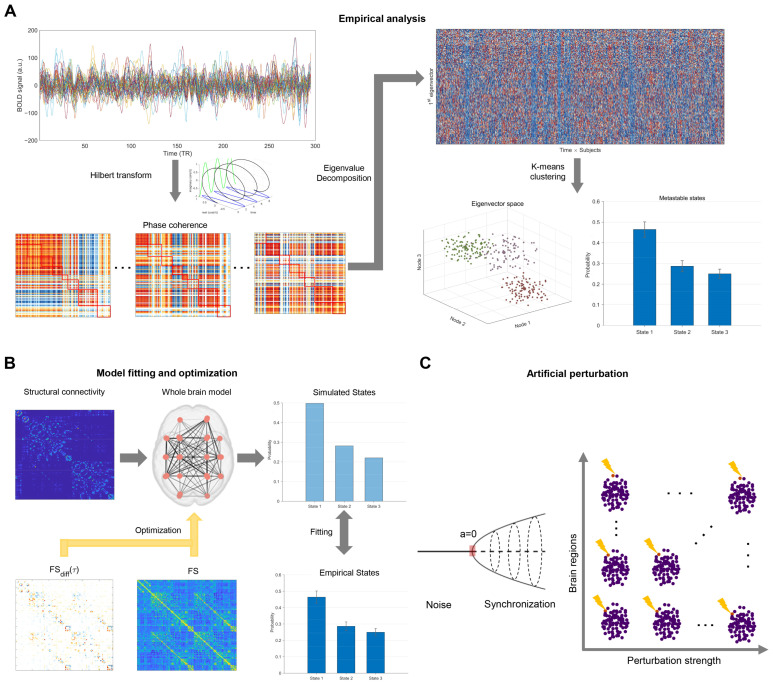
Schematic diagram of the brain state analysis, dynamic modeling, and perturbation framework: (**A**) The Leading Eigenvector Dynamics Analysis was applied to investigate brain states. The regional BOLD signals were filtered (0.01–0.08 Hz) and Hilbert-transformed. Phase coherence matrices were calculated at each time point. The leading eigenvectors were extracted through eigenvalue decomposition and concatenated across time, subjects, and groups. Finally, the k-means clustering algorithm was applied to derive representative brain states and compute the probability of metastable states. (**B**) Fitting and optimization of the whole-brain model. Whole-brain network models were constructed based on structural connectivity. Optimal fitting was achieved by adjusting the global coupling strength to minimize the Jensen-Shannon (J-S) distance between empirical and simulated states. Functional connectivity and difference in time-shifted connectivity guided model optimization and effective connectivity (EC) estimation. (**C**) Perturbation framework. Perturbations were modeled as changes in the local bifurcation parameters, driving regional activity into the noisy regime or synchronization regime. Different perturbation strengths were applied to various brain regions in silico to examine transitions between states.

As shown in Figure 1A, we used the Leading Eigenvector Dynamics Analysis (LEiDA, Oxford, UK) method to perform empirical analysis on functional data [14]. BOLD time series were filtered between 0.01 and 0.08 Hz and Hilbert-transformed to derive phase time series. At each time point, the phase coherence matrix (dFC, N × N dimension) was calculated using cosine similarity, quantifying the synchrony level between brain regions,

(1) $$\mathrm{dFC}\left(i,j,t\right)=\cos\left(\theta \left(i,t\right)-\theta \left(j,t\right)\right).$$

$\theta \left(i,t\right)$ represents the instantaneous phase of the BOLD signal at node *i* and time *t*. The dFC = 1, −1, 0 indicate in-phase, anti-phase, and orthogonal relationships between two brain regions, respectively. Eigenvalue decomposition was applied to reduce the dimensionality of dFC and derive the leading eigenvector ($V_{1}$, N × 1 dimension). The leading eigenvectors preserved the main connectivity patterns, enhanced the signal-to-noise ratio, and captured node-level contributions to brain states [14]. We concatenated these vectors and obtained a 116 × 295 × 88 matrix (regions × time × subjects), representing phase synchrony across regions and time for all subjects in the HCT and CUD groups.

K-means clustering was applied to all leading eigenvectors (295 × 88) with city-block distance and 100 replicates to identify the group-level metastable brain states (cluster centroids) and individual state time series. The outer product of each centroid represented the main functional connectivity patterns of its corresponding states. Based on the individual state series, we calculated the probability of occurrence in each state for each subject, generating the Probabilistic Metastable Substate Spaces (PMS). The PMS enabled us to identify state differences between HCT and CUD. We conducted the above analysis for K = 3 to 8, and then selected the smallest K that showed significant group differences, as presented in the main text. Following previous research [49], we calculated the Pearson correlation between the positive values of the centroids (with negative values set to 0) and both the Yeo 7 networks and subcortical structures to explore the potential associations between brain states and network functions [62]. We also investigated the potential associations between the subject-level state probabilities in CUD and addiction severity indices.

We examined the robustness of state dynamics differences using the SUDMEX-TMS dataset. Since this dataset contains only CUD data, we combined the CUD data from this independent dataset with the HCT data from the main analysis to perform LEiDA. We focused on determining whether the state that exhibited network correlation patterns similar to the significant state identified in the main analysis could replicate the observed differences between the CUD and HCT groups.

### 2.4. Whole-Brain Computational Model

We constructed group-level whole-brain network models to elucidate the mechanisms underlying PMS differences between HCT and CUD. Each brain region was modeled as a Stuart–Landau oscillator [16,20]. Adjusting the local bifurcation parameter from negative to positive allowed the brain activity to transition from noisy fluctuations to oscillations, demonstrating a supercritical Hopf bifurcation. Activities of various brain regions were coupled through SC. The activity of a brain region *i* (*i* = 1–116) was governed by the following equations:

(2) $$\frac{dx_{i}}{dt}=\left[a_{i}-x_{i}^{2}-y_{i}^{2}\right]x_{i}-\omega_{i}y_{i}+G\sum_{j=1}^{N}C_{ij}\left(x_{j}-x_{i}\right)+\sigma w_{i}\left(t\right),$$

(3) $$\frac{dy_{i}}{dt}=\left[a_{i}-x_{i}^{2}-y_{i}^{2}\right]y_{i}+\omega_{i}x_{i}+G\sum_{j=1}^{N}C_{ij}\left(y_{j}-y_{i}\right)+\sigma w_{i}\left(t\right).$$

$x_{i}$ represents the simulated BOLD signal and is disturbed by Gaussian noise $\sigma w_{i}\left(t\right)$. The probability density function of $w_{i}\left(t\right)$ follows the standard Gaussian distribution. σ is the noise amplitude. The bifurcation parameter $a_{i}$ controls the node dynamics: $a_{i}<0$ corresponds to the fixed-point regime with noisy asynchronous activity, while $a_{i}>0$ shifts the node to the limit cycle regime, producing oscillations with angular frequency $\omega_{i}$. $\omega_{i}$ was estimated by $f_{i}\times 2\pi$, where $f_{i}$ was the averaged peak frequency of the empirical BOLD signal at region *i*. $a_{i}$ was set to −0.02 for all regions. This setting aligns with the widely accepted assumption that near-critical fluctuations best capture empirical functional network structure and provide optimal flexibility [63,64]. $C_{ij}$ represents the connection strength between nodes *i* and *j*, and it is scaled by the global coupling strength G. The $C_{ij}$ matrix was normalized so that its maximum value was 0.2, preventing high synchronization [65].

### 2.5. Empirical Fitting of the Whole-Brain Model

The stochastic differential equations were integrated using the Euler–Maruyama numerical scheme [66]. The model was initially run for 3000 s to eliminate instabilities. Simulations were then conducted over a time period equal to the total scanning time of all participants, generating a simulated time series. These generated time series were analyzed using LEiDA to extract leading eigenvectors. Each eigenvector was assigned a label based on its nearest empirical k-means centroid, resulting in a simulated state sequence. Based on these sequences, we calculated the state probabilities and derived the simulated PMS. We quantified the model’s fitting accuracy using the Jensen–Shannon distance (a symmetric variant of Kullback–Leibler divergence) between the simulated and empirical PMS [20,47,50].

(4) $$\mathrm{JS}\left(P_{\mathrm{emp}},P_{\mathrm{sim}}\right)=0.5\left(\sum_{i}P_{\mathrm{emp}}\left(i\right)\ln\left(\frac{P_{\mathrm{emp}}\left(i\right)}{P_{\mathrm{sim}}\left(i\right)}\right)+\sum_{i}P_{\mathrm{sim}}\left(i\right)\ln\left(\frac{P_{\mathrm{sim}}\left(i\right)}{P_{\mathrm{emp}}\left(i\right)}\right)\right),$$

$P_{\mathrm{emp}}\left(i\right)$ and $P_{\mathrm{sim}}\left(i\right)$ are the empirical and simulated occurrence probabilities of state *i*. To obtain the optimal fitting of the whole-brain model, we systematically run simulations under different G, ranging from 0 to 0.5 with increments of 0.01. The working point was determined as the G that minimized the J-S distance between simulated and empirical PMS (Figure 1B).

### 2.6. Optimization of the Whole-Brain Model

For each G value, we optimized SC by updating the potentially inaccurate DWI-derived connections and generated effective connectivity (EC). In this study, we adopted the procedure in [67] to perform optimization, based on FC and time-shifted FC (FC(τ)). We transformed all values in FC and time-shifted FC into mutual information measures (FS and FS(τ)) so that the optimization only handles positive values [67]. The difference (${\mathrm{FS}}_{\mathrm{diff}}\left(\tau \right)$) between the forward and reversal versions of FS(τ) is in line with the INSIDEOUT framework, measuring the thermodynamic nonreversibility (NR) [68]. FS and ${\mathrm{FS}}_{\mathrm{diff}}\left(\tau \right)$ are given by:

(5) $${\mathrm{FC}}_{ij}=\langle x_{i}\left(t\right),x_{j}\left(t\right)\rangle$$

(6) $${\mathrm{FS}}_{ij}=-\frac{1}{2}\log\left(1-{\mathrm{FC}}_{ij}^{2}\right)$$

(7) $${\mathrm{FC}}_{\mathrm{forward},ij}\left(\tau \right)=\langle x_{i}\left(t\right),x_{j}\left(t+\tau \right)\rangle$$

(8) $${\mathrm{FS}}_{\mathrm{forward},ij}\left(\tau \right)=-\frac{1}{2}\log\left[1-{\mathrm{FC}}_{\mathrm{forward},ij}{\left(\tau \right)}^{2}\right]$$

(9) $${\mathrm{FC}}_{\mathrm{reversal},ij}\left(\tau \right)=\langle x_{i}^{r}\left(t\right),x_{j}^{r}\left(t+\tau \right)\rangle$$

(10) $${\mathrm{FS}}_{\mathrm{reversal},ij}\left(\tau \right)=-\frac{1}{2}\log\left[1-{\mathrm{FC}}_{\mathrm{reversal},ij}{\left(\tau \right)}^{2}\right]$$

(11) $${\mathrm{FS}}_{\mathrm{diff},ij}\left(\tau \right)={\mathrm{FS}}_{\mathrm{forward},ij}\left(\tau \right)-{\mathrm{FS}}_{\mathrm{reversal},ij}\left(\tau \right)$$

$x_{i}\left(t\right)$ and $x_{j}\left(t\right)$ represent the BOLD time series of regions *i* and *j*. $x_{i}^{r}\left(t\right)$ and $x_{j}^{r}\left(t\right)$ are the corresponding reverse backward time series by flipping the sequence order. Following previous studies [67,69], we selected *τ* = 2 s. The Pearson correlation coefficient between time series is denoted by ⟨ ⟩. ${\mathrm{FC}}_{\mathrm{forward},ij}\left(\tau \right)$ and ${\mathrm{FC}}_{\mathrm{reversal},ij}\left(\tau \right)$ represent forward and reversal time-shifted correlation. ${\mathrm{FS}}_{\mathrm{forward},ij}\left(\tau \right)$ and ${\mathrm{FS}}_{\mathrm{reversal},ij}\left(\tau \right)$ are their mutual-information transformed version.

The optimization process employed a gradient descent algorithm to minimize the distance between empirical and simulated versions of FS and ${\mathrm{FS}}_{\mathrm{diff}}\left(\tau \right)$:

(12) $$G_{ij}=G_{ij}+\epsilon \left(FS_{ij}^{\mathrm{empirical}}-FS_{ij}^{\mathrm{model}}\right)-\epsilon^{\prime }\{[{\mathrm{FS}}_{\mathrm{forward},\mathrm{ij}}^{\mathrm{empirical}}\left(\tau \right)\;-FS_{\mathrm{reversal},ij}^{\mathrm{empirical}}\left(\tau \right)]-\left[{\mathrm{FS}}_{\mathrm{forward},\mathrm{ij}}^{\mathrm{model}}\left(\tau \right){-\mathrm{FS}}_{\mathrm{reversal},\mathrm{ij}}^{\mathrm{model}}\left(\tau \right)\right]\}.$$

$G_{ij}$ represents the EC between regions *i* and *j*. The initial value is the empirical group-level structural connectivity derived from DWI data. The algorithm is restricted to updating only pre-existing connections. However, given the known limitations of tractography in capturing interhemispheric connectivity, an exception was made for homologous connections between corresponding regions across hemispheres. The learning rates ϵ and $\epsilon^{\prime }$ were set to 0.001 and 0.002, respectively. In each iteration, we calculated the model results using the generated time series where the length of each series was equal to the total scanning time of all participants. The connectivity matrix was normalized to a maximum value of 0.2 after each iteration. The process continued until the algorithm converged.

The predictive accuracy of a model relies on how well it describes empirical data [70,71,72]. Compared to single-constraint optimization using only FS, multi-constraint optimization could provide a more comprehensive and accurate characterization of brain activity, potentially yielding different perturbation effects and optimal target regions. Specifically, FS quantifies spatial patterns of inter-regional coordination by capturing statistical dependencies in neural activity across brain regions. By integrating FS into the computational framework, the model achieves higher precision in simulating undirected functional couplings between regions, potentially improving its predictive capacity for neural activity. Critically, the brain operates as a non-equilibrium thermodynamic system, where information flow is inherently asymmetric over time. NR reflects hierarchical processing and causal interactions in the brain by leveraging the arrow of time [68,69,73]. Incorporating the NR constraint allows the model to capture the unidirectional nature of neural signal processing, making the model dynamics more closely aligned with the empirical operational mechanisms of the brain. Although this study focused on exploring perturbation effects in whole-brain models with multi-constraint optimization, we provided perturbation results based on FS optimization in the Supplementary Materials for direct comparison.

The group-level EC matrix integrated information from SC, FC, and NR. Its connection pattern indicated the brain’s intrinsic functional organization and hierarchical structure. The total information flow of node *i* was calculated as

(13) $$G_{\mathrm{total}}\left(i\right)=\sum_{j}G_{ij}+\sum_{j}G_{ji}$$

Following prior work [67], we used $G_{\mathrm{total}}$ to investigate functional organization differences between HCT and CUD conditions.

### 2.7. Artificial Perturbation Protocol

Using the model that optimally fitted empirical PMS, we investigated possible transitions between HCT and CUD conditions through perturbation analysis (Barcelona, Spain) [20,49]. We implemented the single-region perturbation protocol with varying perturbation strengths. The perturbation strength was defined as the change in the local bifurcation parameter for the targeted region, ranging from −0.3 to 0.05 (steps of 0.01) for CUD to HCT transitions and from −0.15 to 0.2 (steps of 0.01) for HCT to CUD transitions. Positive perturbations drove the brain region into a synchronization regime with oscillations, while negative perturbations placed it in a noisy regime with low fluctuations [63].

To ensure optimal results, we conducted systematic explorations across different perturbation strengths and sites (Figure 1C). Each trial was repeated 10 times to minimize the influence of random noise. The perturbation duration matched the total scanning time of all participants within each group. The perturbed PMS was derived by analyzing the perturbed time series with the empirical cluster centroids following methods in previous subsections. We quantified the perturbation effects by calculating the Jensen–Shannon (J-S) distance between the perturbed and empirical PMS, where smaller distances indicated more effective state transitions.

All datasets, software, and algorithms used in this study are summarized in Table 1.

### 2.8. Statistical Analysis

Multivariate analysis of variance (ANOVA) was performed to assess the overall differences in state probability distributions between HCT and CUD groups, with the group as the independent variable, all K state probabilities as dependent variables, and age, sex, education, and average framewise displacement as covariates. Following this omnibus test, univariate ANOVA was also conducted to compare single-state probabilities between groups with the same covariates as post hoc analysis. Multiple comparisons were corrected using the False Discovery Rate (FDR) method with the Benjamini–Hochberg procedure across states in each cluster number K [47,50].

The 95% confidence intervals of Pearson’s correlation coefficients between brain states and both Yeo 7 functional networks and subcortical structures were calculated using the Fisher z-transformation. Multiple comparisons were corrected for *p* values of correlations using the FDR method with the Benjamini–Hochberg procedure across the number of functional networks.

The potential associations between the subject-level state probabilities in CUD and the addiction severity indices were explored using Spearman’s partial correlation, controlling age, sex, education, and average framewise displacement as covariates. The 95% confidence intervals for these Spearman correlation coefficients were computed using the bootstrap method with 1000 bootstrap replicates. The differences between the results of optimization methods were compared using paired *t*-tests. The statistical significance level was set at *p* < 0.05.

## 3. Results

### 3.1. Empirical Analysis

**Figure 2:**
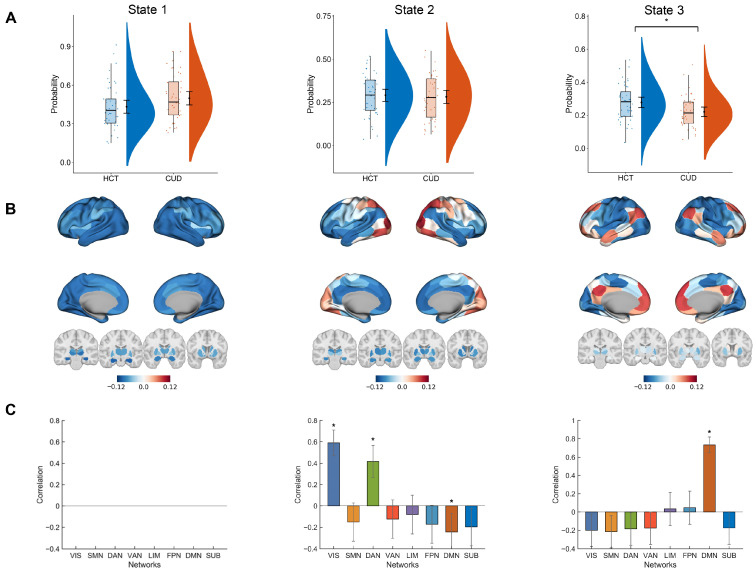
LEiDA results and group comparison: (**A**) Raincloud plots showing state occurrence probabilities across groups. Violins (right portions of subplots) represent data distributions. Nodes and error bars next to violins represent mean values and 95% confidence intervals. Box plots (left portions of subplots) show the median (middle lines), interquartile ranges (box length), and 1.5 × quartile ranges (whiskers). Individual data points are jittered. The multivariate ANOVA test controlling age, sex, education, and mean framewise displacement indicated significant differences in the state probability distributions between HCT and CUD groups (F(2,81) = 3.67, *p* = 0.030). Post hoc analyses using univariate ANOVA showed a significant difference in state 3 (F(1,82) = 7.42, corrected *p* = 0.024). (**B**) Three representative dynamic states (eigenvector centroids) rendered on the cortical surface and subcortical regions. (**C**) Pearson correlations between states and both the Yeo 7 networks and subcortical structures. State 1 showed global synchronization without network-specific correlations. State 2 correlated positively with VIS (r = 0.59, 95% CI [0.47, 0.7], corrected *p* < 0.001) and DAN (r = 0.42, 95% CI [0.27, 0.57], corrected *p* < 0.001) and negatively with DMN (r = −0.24, 95% CI [−0.41, −0.07], corrected *p* = 0.022). State 3 showed a predominant positive correlation with DMN (r = 0.73, 95% CI [0.64, 0.82], corrected *p* < 0.001). VIS: visual network, SMN: sensorimotor network, DAN: dorsal attention network, VAN: ventral attention network, LIM: limbic network, FPN: frontoparietal network, DMN: default mode network, SUB: subcortical structures. *p*-values were FDR-corrected using the Benjamini–Hochberg procedure with * representing corrected *p* < 0.05.

In this subsection, we employed LEiDA to analyze empirical functional data. The aim was to identify meaningful brain states and examine dynamic differences between HCT and CUD groups. We selected K = 3 as the cluster number for the main analysis, as it is the smallest K that can distinguish between groups. The multivariate ANOVA analysis shows a significant difference in the state probability distributions between HCT and CUD groups (F(2,81) = 3.67, *p* = 0.030), with age, sex, education, and average framewise displacement as covariates. The performance of other K values is shown in Figure S1, where K = 4, 5, and 7 also exhibited significant group differences. Figure 2A shows the comparison of single-state probability between the HCT and CUD groups using univariate ANOVA for post hoc analyses, controlling age, sex, education, and average framewise displacement. State 3 occurred significantly more frequently in HCT compared to CUD (F(1,82) = 7.42, corrected *p* = 0.024). State 1 showed a trend toward higher frequency in CUD, although not significant (F(1,82) = 3.68, corrected *p* = 0.087). State 2 demonstrated comparable probabilities between groups (F(1,82) = 0.08, corrected *p* = 0.780).

To understand the spatial patterns of states, we mapped them onto the cortical surface and subcortical structures (Figure 2B). The states showed two different functional communities (red or blue) depending on the sign of their elements (positive or negative). Furthermore, we correlated state patterns with the Yeo 7 networks and subcortical structures to characterize their potential functional features [62] (Figure 2C). State 1 showed widely negative elements with the highest occurrence probability. It represented a global synchronization state and showed no correlation with the seven networks. State 2 showed positive values mainly in regions like the primary visual cortex and superior parietal lobule. It showed significant positive correlations with VIS (r = 0.59, 95% CI [0.47, 0.7], corrected *p* < 0.001) and DAN (r = 0.42, 95% CI [0.27, 0.57], corrected *p* < 0.001), and a negative correlation with DMN (r = −0.24, 95% CI [−0.41, −0.07], corrected *p* = 0.022), suggesting involvement in external information processing. State 3 showed activation in the medial prefrontal cortex, posterior cingulate, angular gyrus, and lateral temporal cortex, with strong positive DMN correlation (r = 0.73, 95% CI [0.64, 0.82], corrected *p* < 0.001). This correlation pattern suggests that state 3 is task-negative, representing internal thought and self-reflection.

**Figure 3:**
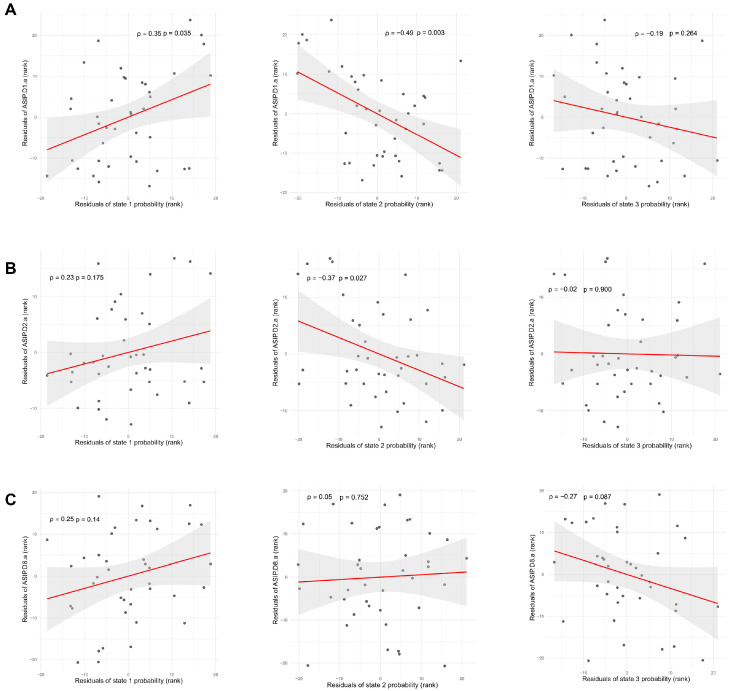
The associations between brain states and addiction severity indices derived using Spearman’s partial correlation controlling age, sex, education, and average framewise displacement. Data points are residuals of ranked values after removing covariates: (**A**) ASIP.D1.a (Alcohol use in the past thirty days) was significantly correlated with state 1 probability (ρ = 0.35, 95% CI [−0.09, 0.64], *p* = 0.035) and state 2 probability (ρ = −0.49, 95% CI [−0.74, −0.06], *p* = 0.003). ASIP.D1.a was not correlated with state 3 probability (ρ = −0.19, 95% CI [−0.53, −0.20], *p* = 0.264). (**B**) ASIP.D2.a (Alcohol to intoxication in the past thirty days) was significantly correlated with state 2 probability (ρ = −0.37, 95% CI [−0.64, −0.02], *p* = 0.027). ASIP.D2.a was not correlated with state 1 (ρ = 0.23, 95% CI [−0.14, 0.54], *p* = 0.175) and state 3 probability (ρ = −0.02, 95% CI [−0.37, −0.37], *p* = 0.900). (**C**) ASIP.D8.a (Cocaine in the past thirty days) showed a tendency toward a negative correlation with the state 3 probability, but the association is not significant (ρ = −0.27, 95% CI [−0.60, 0.09], *p* = 0.087). ASIP.D8.a was not correlated with state 1 (ρ = 0.25, 95% CI [−0.08, 0.54], *p* = 0.140) and state 2 probability (ρ = 0.05, 95% CI [−0.28, 0.39], *p* = 0.752).

In the replication analysis, we found the DMN-related states showing significant differences between HCT and CUD groups across clustering number K = 5–8, validating the robustness of the pattern (Figure S2). Additionally, we explored the relations between state probabilities and addiction severity indices using Spearman’s partial correlation, with age, sex, education, and average framewise displacement as covariates (Figure 3). The results showed that the state 2 probability was negatively correlated to both alcohol use (ρ = −0.49, 95% CI [−0.74, −0.06], *p* = 0.003) and alcohol consumption to intoxication (ρ = −0.37, 95% CI [−0.64, −0.02], *p* = 0.027) over the past 30 days. The state 1 probability positively correlated with any alcohol use over the past 30 days (ρ = 0.35, 95% CI [−0.09, 0.64], *p* = 0.035). The state 3 probability exhibited a negative correlation trend with cocaine use in the past 30 days; however, the association was not significant (ρ = −0.27, 95% CI [−0.60, 0.09], *p* = 0.087).

### 3.2. Model Fitting and Optimization

In this subsection, we constructed whole-brain network models to reproduce the observed state probabilities in both groups. We adjusted G and optimized inter-regional connections to achieve the optimal fit. Model performance was evaluated using the J-S distance between the empirical and simulated state distributions, where smaller distances indicate a better fit. We also showed that FS and ${\mathrm{FS}}_{\mathrm{diff}}\left(\tau \right)$ optimization imposed more constraints on the model than FS optimization alone, potentially offering a more accurate characterization of the underlying functional organization. The constructed models also established a reliable foundation for subsequent regional perturbation analyses.

**Figure 4:**
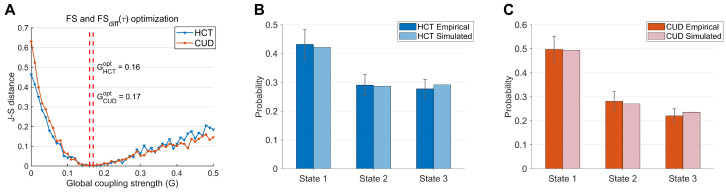
Whole-brain model performance: (**A**) J-S distance between empirical and simulated brain states as a function of global coupling strength G. The optimal fits were found at G = 0.16 (HCT) and G = 0.17 (CUD). At each G value, the whole-brain model was optimized using FS and ${\mathrm{FS}}_{\mathrm{diff}}\left(\tau \right)$. (**B**) The occurrence probabilities of empirical states and the corresponding best-fit states for HCT. (**C**) The occurrence probabilities of empirical states and the corresponding best-fit states for CUD. Error bars represent 95% confidence intervals. Bar plots summarize the group-level empirical and model performance metrics. Light-colored data represent simulated state probability dynamics aggregated over time intervals equivalent to scan durations of all individuals.

Figure 4A shows the J-S distance for the HCT and CUD as a function of G. The optimal fits were found at G = 0.16 with a J-S distance 0.0005 for HCT and at G = 0.17 with a J-S distance 0.0007 for CUD. The scenario where G = 0 (no inter-regional interactions) is considered as a benchmark. Figure S3 presents the results of comparisons between model optimizations conducted under benchmark conditions and those performed with the best-fitted G value, demonstrating a statistically significant improvement over the benchmark. For the HCT group, the simulated state probabilities (state 1: 0.422, state 2: 0.287, state 3: 0.292) closely matched empirical values (state 1: 0.432, 95% CI [0.380, 0.484]; state 2: 0.291, 95% CI [0.254, 0.328]; state 3: 0.278, 95% CI [0.245, 0.311]) (Figure 4B). For the CUD group, the simulated state probabilities (state 1: 0.494, state 2: 0.270, state 3: 0.236) were also well aligned with empirical values (state 1: 0.498, 95% CI [0.444, 0.552]; state 2: 0.282, 95% CI [0.242, 0.322]; state 3: 0.221, 95% CI [0.192,0.250]) (Figure 4C). Additional analyses using only FS-based optimization yielded similar results, indicating that including ${\mathrm{FS}}_{\mathrm{diff}}\left(\tau \right)$ did not significantly alter model fitting performance (Figure S4).

**Figure 5:**
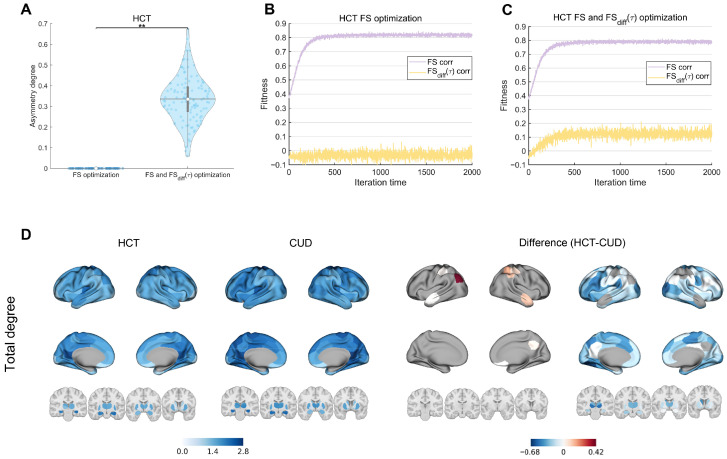
Whole-brain model optimization revealed functional configurations in HCT and CUD from a causal perspective: (**A**) Violin plots showing the asymmetry degree of EC following FS optimization alone versus FS and ${\mathrm{FS}}_{\mathrm{diff}}\left(\tau \right)$ optimization. Colored dots represent subject-level measures, box limits correspond to the 25th and 75th percentiles, horizontal lines mark the medians, white dots represent the mean, and whiskers cover the 1.5 interquartile range. The comparison was made using a paired *t*-test. ** represents *p* < 0.001. (**B**) The evolution of empirical-simulated correlations for FS (purple) and ${\mathrm{FS}}_{\mathrm{diff}}\left(\tau \right)$ (yellow) during FS optimization. (**C**) The evolution of empirical-simulated correlations for FS (purple) and ${\mathrm{FS}}_{\mathrm{diff}}\left(\tau \right)$ (yellow) during FS and ${\mathrm{FS}}_{\mathrm{diff}}\left(\tau \right)$ optimization. (**D**) (left) The total information flow of each node rendered on the cortical surface and subcortical structures. (right) Positive and negative differences in total information flow between states (HCT-CUD).

The FS and ${\mathrm{FS}}_{\mathrm{diff}}\left(\tau \right)$ optimization induced a stronger asymmetry degree of EC in the HCT condition (t(115) = −33.3, *p* < 0.001) (Figure 5A). In contrast, FS optimization showed little asymmetry, aligning with previous findings [67]. EC matrices optimized using FS and ${\mathrm{FS}}_{\mathrm{diff}}\left(\tau \right)$ are presented in Figure S5. Figure 5B,C show the evolution of model fitness under different optimization approaches. Both approaches achieved good FS fit as iterations progressed. The ${\mathrm{FS}}_{\mathrm{diff}}\left(\tau \right)$ correlation fluctuated near 0 under FS-only optimization but exceeded 0.1 under combined optimization. Although the correlation was not so high, it remained significantly higher than that obtained by FS-only optimization, indicating that the model captured unique NR characteristics. The result suggests that NR contains information distinct from FC, and the model optimization must include ${\mathrm{FS}}_{\mathrm{diff}}\left(\tau \right)$ to replicate the NR property [67]. The combined optimization enabled the model to explain and capture more empirical data properties compared to FS-only optimization, potentially leading to new insights into optimal perturbation targets. The CUD model showed similar results (Figures S6 and S8). Additionally, we observed that ${\mathrm{FC}}_{\mathrm{phase}}$ and ${\mathrm{FS}}_{\mathrm{forward}}\left(\tau \right)$ were also optimized during FS optimization alone, further highlighting the uniqueness of ${\mathrm{FS}}_{\mathrm{diff}}\left(\tau \right)$ (Figure S7).

The EC generated by the whole-brain model reflects causal information flow between brain regions. We use the total information flow ($G_{\mathrm{total}}$) to explore functional organization differences between HCT and CUD (Figure 5D). We found that the CUD showed a lower degree in areas such as the left inferior parietal lobule in DMN, right temporal region in DMN, and right intraparietal sulcus in FPN and DAN. This result implies impaired functional coordination and information flow in CUD patients, potentially underlying deficits in emotional regulation and attention control [11,74]. Most other regions showed an increased total degree in CUD compared to HCT, indicating excessive inter-regional information flow.

### 3.3. Mediating Transitions from HCT to CUD Through Perturbations

In this subsection, we investigated how perturbations induced transitions of brain states from HCT to CUD. The model was fitted and optimized using functional data and structural connectivity from the HCT group. We applied single-region perturbations with varying strengths (−0.15 to 0.2) to the model, where positive values indicated synchronization protocols and negative values represented noise protocols. The optimal perturbation corresponded to the minimal J-S distances between the perturbed model’s PMS and the empirical CUD PMS. This analysis contributed to understanding the potential pathogenic mechanisms underlying addiction behaviors.

**Figure 6:**
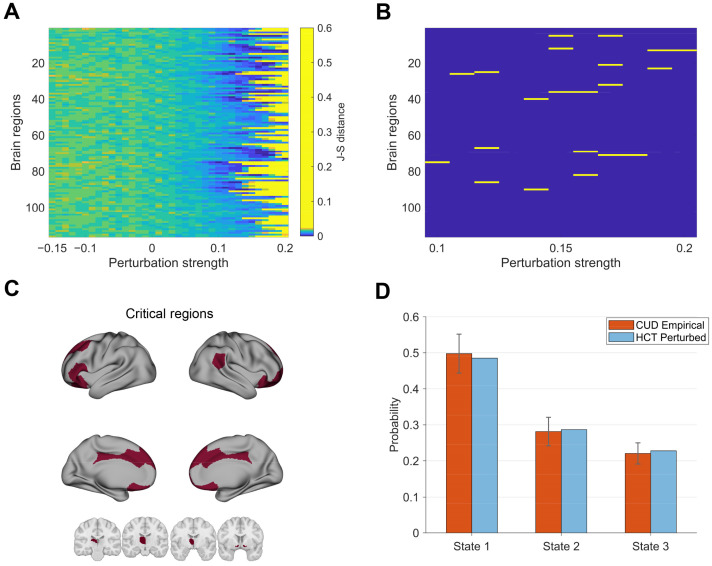
Transitions from HCT to CUD: (**A**) J-S distances between the empirical CUD states and perturbed HCT states across varying perturbation strengths (−0.15 to 0.2) and targeted regions. (**B**) Regions with the highest perturbation efficacy were identified by selecting the top 0.5% areas with minimal J-S distances. (**C**) Critical brain regions in (**B**) rendered on the cortical surface and subcortical regions. (**D**) Comparison of occurrence probabilities between the states induced by stimulating the right dorsal prefrontal cortex and the empirical CUD states. Bar plots summarize the group-level empirical and model performance metrics. Light-colored data represent model-stimulated state probability dynamics aggregated over time intervals equivalent to scan durations of all individuals.

Figure 6A shows that noise protocols generally increased the J-S distances, indicating poor alignment with CUD states. In contrast, positive perturbation strengths could decrease the J-S distances. We observed minimal J-S distances (deep blue) within the 0.1–0.2 perturbation range, suggesting optimal reproduction of the pathological condition. Further increasing the perturbation strength yielded rapid increased J-S distance (light yellow) across most regions, indicating poor fit. Different brain regions showed distinct responses to perturbation at fixed strengths. To identify regions with optimal perturbation effects, we applied a threshold approach to the data presented in Figure 6A and selected perturbation configurations with the top 0.5% (n = 21) minimal J-S distances (Figure 6B). By comparing the results of perturbation configurations in Figure 6B with corresponding benchmarks (perturbation strength = 0), we showed optimal perturbations significantly drove the system toward CUD (Figure S9). We further visualized these regions on the cortical surface and subcortical areas in Figure 6C. Critical regions are located in the nucleus accumbens (bilateral), thalamus (left), ventral prefrontal cortex (bilateral), dorsal prefrontal cortex (dPFC, bilateral), posterior cingulate cortex (bilateral), orbitofrontal cortex (bilateral), frontal medial cortex (bilateral), frontal pole (right), and inferior parietal lobule (right), associated with high-order cognitive networks DMN, LIM, and FPN. Figure 6D demonstrates the result of right dPFC perturbation, which effectively induced transitions from HCT to CUD-like dynamics. Specifically, this perturbation resulted in reduced occupancy probabilities of state 3 compared to the baseline HCT states previously illustrated in Figure 2A.

We provided the perturbation effects based on FS-only optimization in Figure S10. By comparing optimal perturbation configurations, we found that the combined optimization introduced new potential perturbation targets and strengths. The results were robust when using an alternative threshold criterion (1%) (Figure S11). We also compared perturbation asymmetry between the two optimization approaches. Specifically, the perturbation asymmetry was calculated as the mean absolute difference in J-S distances between homologous perturbation regions across the strength range. The combined FS and ${\mathrm{FS}}_{\mathrm{diff}}\left(\tau \right)$ optimization showed significantly higher asymmetry, enabling the distinction between left and right hemisphere perturbations (Figure S12).

### 3.4. Promoting Transitions from CUD to HCT Using Perturbations

Finally, we explored the possibility of brain-state transitions from CUD to HCT using external perturbations. The model was fitted and optimized using functional and structural data of the CUD group. We systematically altered the perturbation regions and strengths to identify the optimal perturbations that minimize the J-S distance between the perturbed model’s PMS and the empirical HCT PMS. We set the strength range between −0.3 and 0.05. The lower bound was chosen following previous research [49], while the upper bound was selected to maintain the same range length as in Figure 6. Perturbation results for strengths larger than 0.05 are provided in Figure S13. This analysis provides valuable insights for developing stimulation-based treatments.

**Figure 7:**
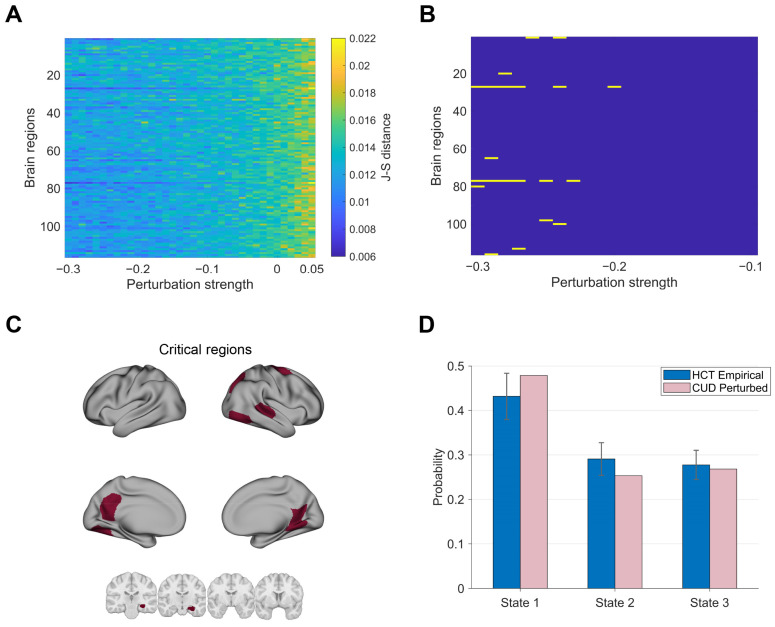
Transitions from CUD to HCT: (**A**) J-S distances between the empirical HCT states and perturbed CUD states across varying perturbation strengths (−0.3 to 0.05) and targeted regions. (**B**) Regions with the highest perturbation efficacy were identified by selecting the top 0.5% areas with minimal J-S distances. (**C**) Critical brain regions in (**B**) rendered on the cortical surface and subcortical regions. (**D**) Comparison of occurrence probabilities between the states induced by stimulating the right retrosplenial cortex and the empirical HCT states. Bar plots summarize the group-level empirical and model performance metrics. Light-colored data represent model-stimulated state probability dynamics aggregated over time intervals equivalent to scan durations of all individuals.

The synchronization protocol increased the J-S distances, resulting in poor alignment with the HCT dynamics (Figure 7A). Instead, increasing noisy perturbation strengths in specific brain regions reduced the J-S distances and drove the system toward the HCT condition. We observed minimal J-S distances (deep blue) within the strength from −0.3 to −0.1. To identify critical brain regions, we selected the top 0.5% of perturbation configurations (21 configurations) with minimal J-S distances (Figure 7B). By comparing the results of perturbation configurations in Figure 7B with corresponding benchmarks (perturbation strength = 0), we found optimal perturbations significantly drove the system back to HCT (Figure S9). We further visualized these regions in Figure 7C. Critical regions were mainly located in the hippocampus (right), posterior cingulate cortex (left), retrosplenial cortex (RSC, bilateral), precentral cortex (right), superior parietal lobule (right), and visual areas (bilateral), involving functional networks VIS, DAN, and DMN. Figure 7D illustrates the optimal perturbation effect of the right RSC as an example. This perturbation effectively drove the system back to health, with an increased probability of state 3 relative to the CUD state presented in Figure 2A.

Notably, Figure 7B demonstrates that the RSC maintained optimal fit across various strength levels, whereas the optimal regions shown in Figure S10 with FS-only optimization appeared more dispersed. This result may suggest that the combined optimization approach may help identify reliable and robust stimulation targets. Analysis of perturbation using a 1% threshold yielded similar results (Figure S11). The combined FS and ${\mathrm{FS}}_{\mathrm{diff}}\left(\tau \right)$ optimization also showed significantly higher asymmetry of perturbation effects (Figure S12).

## 4. Discussion

In this study, we combined empirical analysis and computational modeling to investigate brain dynamics and state transitions in CUD. First, we applied LEiDA to extract representative brain states with associated occurrence probabilities and distinguished HCT and CUD groups. We then reproduced the empirical state probabilities through fitting and optimizing whole-brain network models. The constructed models captured the brain dynamics under different conditions in silico, establishing a foundation for perturbation analyses. Finally, we perturbed different brain regions with varying strengths to systematically investigate transitions between brain states. Our results revealed that positive perturbations (synchronization protocol) facilitated transitions toward the CUD condition, while negative perturbations (noise protocol) reshaped brain dynamics back to health. We also identified the critical brain regions most effective at inducing transitions between healthy and pathological conditions.

We found that the CUD group showed significantly reduced probability in state 3 compared to HCT. This state was characterized by local coordination among the medial prefrontal cortex, posterior cingulate cortex, angular gyrus, and lateral temporal cortex. Analysis of Yeo 7 networks revealed that this state strongly overlapped with DMN, which is associated with task-negative functions including self-reflection, episodic memory, and emotion processing [75]. The low frequency of this state suggests impaired DMN function, potentially manifesting as decreased inhibitory control, compromised emotional processing, and enhanced drug craving [11]. Previous task-based fMRI research has identified altered dynamic connectivity in the DMN as a key feature of cocaine dependence [76]. These connectivity alterations likely interact with brain-state dynamics to influence addiction behaviors. Our findings also align with existing literature demonstrating DMN-related state abnormalities in substance use disorders. Studies have shown reduced DMN-related state persistence in CUD patients using coactivation pattern analyses [24], and decreased fraction time and dwell time in DMN-related states among patients with opioid and alcohol use disorders [77]. Our results extend these observations by demonstrating reduced DMN-state frequency from a phase-coherence perspective.

Our analysis also revealed relationships between brain-state probabilities and addiction severity indices. We observed that state 2 was negatively related to alcohol use and intoxication frequency over the past 30 days, and that state 1 was positively associated with alcohol use frequency over the past 30 days. The probability of state 3 showed a trend toward a negative association with cocaine use. These distinct correlation patterns suggest systematic alterations in brain dynamics, potentially reflecting neuropathological adaptations to substance addiction [24]. The predominance of state 1 (characterized by global synchronization) in severe addiction individuals also resembled neural dynamics observed in other psychiatric conditions such as schizophrenia [49].

The generated EC causally revealed the hierarchical functional organization in terms of inter-regional communication patterns. We observed higher total information flow in most brain regions in CUD compared to HCT. The increased flow may reflect compensatory mechanisms for functional deficits [78] or addiction-related reinforcement learning processes [43]. In line with our results, recent studies have found that CUD patients exhibited increased connectivity within and among multiple networks, suggesting facilitated information transfer [25]. We also identified impaired information flow in specific regions of FPN and DAN, including the left inferior parietal lobule, right temporal region, and right intraparietal sulcus. The low computations in these regions may underlie characteristic cognitive deficits, emotional dysregulation, and impulse control difficulties observed in CUD [11,74].

Using the constructed models, we examined state transitions induced by perturbations. By systematically varying the perturbation strengths and targeted regions, we first identified critical brain regions driving transitions from HCT to the CUD. We found that appropriate positive perturbations reduced the J-S distance between the perturbed states and empirical CUD states, indicating that the abnormal dynamics in CUD may result from increased intrinsic regional activity. The result is in line with the view that cocaine cravings induce distributed brain activation, potentially contributing to the reinforcement of addiction [79]. We identified critical regions located in the nucleus accumbens, thalamus, ventral prefrontal cortex, dorsal prefrontal cortex, posterior cingulate cortex, orbitofrontal cortex, frontal medial cortex, frontal pole, and inferior parietal lobule, primarily within higher order networks: DMN, LIM, and FPN. Stimulating these regions led to an upward trend in state 1 probability and a decrease in state 3 probability. These findings align with previous research delineating the prefrontal cortex, posterior cingulate cortex, and striatum (including nucleus accumbens) as key regions in addictive behaviors [80]. Additionally, the nucleus accumbens and thalamus play crucial roles in motivation and drug cue processing, with heightened activity in these regions linked to lower abstinence rates [81,82,83,84]. Increased activity in subregions of the ventromedial prefrontal cortex has been associated with failed cocaine craving suppression [85]. The left dorsolateral prefrontal cortex showed stronger activation in CUD patients compared to healthy controls [86], further supporting our results.

Finally, we demonstrated that artificial perturbations could induce transitions from CUD to HCT. We also identified optimal areas to rebalance the underlying brain dynamics in CUD patients. Results showed that negative perturbations effectively drove the system back to health, aligning with the perturbation results used to restore functions in schizophrenia patients [49]. Previous research has also shown that hub nodes with important functions in healthy subjects required more negative values of bifurcation parameters [87]. Regions within the VIS, DAN, and DMN exhibited the highest perturbation efficacy. Specifically, critical regions mainly included the hippocampus, posterior cingulate cortex, retrosplenial cortex, precentral cortex, superior parietal lobule, and visual areas. The hippocampus, critical for memory and contextual associations, influences the recall of drug-related cues [88,89,90,91]. Modulating hippocampal activity may disrupt craving-related neural circuits. Regions within the DMN and DAN showed significant alterations in addiction, leading to attention deficits, impaired emotional regulation, and self-awareness [11]. The retrosplenial cortex demonstrated optimal responses across wide perturbation strengths. The result aligns with a longitudinal rat study showing significant connectivity changes in the retrosplenial cortex during addiction, highlighting its potential as a stimulation target for CUD treatment [38]. Additionally, cocaine users exhibited abnormal activation in visual areas [92,93], and noisy perturbations to these regions help reduce hyper-reactivity to addiction-related cues. Note that we observed distinct optimal nodes for facilitating transitions to HCT and CUD states. This disparity likely reflects network reorganization resulting from long-term cocaine use, which disrupts both structural and functional brain connectivity. In such dysregulated networks, compensatory mechanisms emerge where alternative nodes assume more critical roles than those initially induced damage. Previous studies have also identified different pathogenic and regulatory nodes [48], in line with our results.

The combined FS and ${\mathrm{FS}}_{\mathrm{diff}}\left(\tau \right)$ optimization showed higher perturbation asymmetry compared to FS-only optimization, aligning with the brain lateralization theory [94,95]. Previous research has demonstrated hemispheric asymmetry in the neural processing of addiction-related impulsivity and craving [96]. Our model may help distinguish the effects of left versus right hemisphere perturbations. Additionally, the combined optimization approach also introduced new potential stimulation targets, which need further validation through artificial stimulation techniques such as TMS.

## 5. Limitations

Our study has several limitations. First, we performed analyses using k-means clustering states and group-averaged structural connectivity, though it was the first application of computational perturbation approaches to investigating CUD. Given significant individual variations in brain dynamics [97], future studies should incorporate individual-level dynamic analyses and modeling. Second, since cocaine use has long-term effects, future research should examine different stages of CUD including acute effects and withdrawal periods. Third, the dynamic model used in this study was not validated via cross-validation, leave-one-out, or hold-out methods, given its focus on characterizing the collective dynamic behavior of brain states and the expensive computational load to perform iterative numerical integration. We changed the global coupling strength and performed model optimization to achieve the best model fit in line with previous research [20,47,50]. Future studies with larger datasets and distributed computing frameworks could further test the generalizability of such models. Fourth, while the SUDMEX-TMS dataset provided an independent replication cohort for validating state dynamics, this dataset exclusively included CUD patients without healthy controls, and the partial validation of combining these data with healthy controls from the main analysis leads to a critical limitation. Future studies should incorporate larger, fully independent cohorts with balanced clinical profiles to strengthen the robustness of state dynamics in CUD. Fifth, while we controlled for demographic variables (e.g., age, sex, education) as covariates in statistical analyses, the SUDMEX-CUD and SUDMEX-TMS datasets exhibit inherent demographic biases. For instance, the male-dominated samples (84% male in SUDMEX-CUD, 85% in SUDMEX-TMS) may influence brain-state dynamics and limit the generalizability of our findings to broader populations. Future studies should validate these results using demographically balanced cohorts with diverse gender, cultural, and socioeconomic backgrounds. Finally, while our perturbation framework provides useful insights, it remains a simplified model of neural activity. Future improvements could include complex perturbation protocols [98], enhanced regional heterogeneity [99], and additional fitting metrics to comprehensively capture the characteristics of empirical data.

## 6. Conclusions

In summary, this study utilized LEiDA to characterize brain-state differences between the HCT and CUD groups, revealing altered DMN-related state dynamics in CUD. Given that empirical stimulation experiments are time-consuming and potentially risky, we adopted a computational approach to explore perturbation effects across multiple parameters. Our goal was not to enforce perfect transitions between conditions but to reveal the efficacy of external perturbations to brain regions. We identified regions for optimal perturbation using the whole-brain model, providing valuable insights into the stimulation-based treatments for CUD in clinical applications.

## Figures and Tables

**Table 1:** Datasets, software, and algorithms used in this study.

Name	Type	Origin	Version	Authors and Citations
SUDMEX-CUD	dataset	the National Institute of Psychiatry in Mexico City	v1.1.2	Diego Angeles-Valdez et al. [51]
SUDMEX-TMS	dataset	the National Institute of Psychiatry in Mexico City	v2.1.0	Diego Angeles-Valdez et al. [52]
fMRIPrep	software	Stanford University, California, United States	24.1.1	Oscar Esteban et al. [53,54]
DSI studio	software	University of Pittsburgh, Pennsylvania, United States	“Hou” version	Fang-Cheng (Frank) Yeh et al. [58,59]
XCP-D	software	University of Pennsylvania, Pennsylvania, United States	0.10.0rc1	Kahini Mehta et al. [55]
QSDR	algorithm	University of Pittsburgh, Pennsylvania, United States	“Hou” version	Fang-Cheng (Frank) Yeh et al. [58]
modified FACT	algorithm	University of Pittsburgh, Pennsylvania, United States	“Hou” version	Fang-Cheng (Frank) Yeh et al. [59]
LEiDA	algorithm	University of Oxford, Oxford, United Kingdom	N/A	Joana Cabral et al. [14]
Modeling and Perturbation framework	algorithm	Universitat Pompeu Fabra, Barcelona, Spain	N/A	Gustavo Deco et al. [20,67]
Euler-Maruyama Numerical Scheme	algorithm	Ochanomizu University, Japan	N/A	Gisiro Maruyama et al. [66]

## Data Availability

The SUDMEX CONN imaging dataset is available at https://openneuro.org/datasets/ds003346 (accessed on 25 January 2025). The SUDMEX TMS imaging dataset is available at https://openneuro.org/datasets/ds003037/versions/2.1.0 (accessed on 25 January 2025).

## Supplementary Materials

The following supporting information can be downloaded at: https://www.mdpi.com/article/10.3390/brainsci15030263/s1, Figure S1: Clustering results for K = 3–8 states. Figure S2: Robust analysis for the DMN-related state across different cluster number K. Probabilities of occurrence were computed and compared using CUD data from the SUDMEX TMS dataset. Figure S3: Comparisons of J-S distance between best fits and the benchmark (G = 0) using a two-sample *t*-test. Figure S4: Whole-brain model performance with FS optimization alone. Figure S5: Effective connectivity generated by the FS and ${\mathrm{FS}}_{\mathrm{diff}}\left(\tau \right)$ optimization. Figure S6: Violin plots of asymmetry degree under two optimization approaches for CUD. Figure S7: The evolution of correlations between empirical and simulated values of ${\mathrm{FC}}_{\mathrm{phase}}$(yellow) and ${\mathrm{FS}}_{\mathrm{forward}}\left(\tau \right)$(purple). Figure S8: The evolution of correlations between empirical and simulated values of FS (purple) and ${\mathrm{FS}}_{\mathrm{diff}}\left(\tau \right)$(yellow) for the CUD model. Figure S9: Comparisons of J-S distance between optimal perturbation configurations (n = 21) and the benchmark results with the same number (perturbation strength = 0) using a two-sample *t*-test. Figure S10: Perturbation results using FS optimization. Figure S11: Sensitive brain regions selected using threshold 1% under different transitions and optimization methods. Figure S12: Perturbation asymmetry of state transitions. Figure S13: Perturbation results using FS and ${\mathrm{FS}}_{\mathrm{diff}}\left(\tau \right)$ optimization. J-S distances between the empirical HCT states and perturbed CUD states across different strengths (0 to 0.2) and regions.
