# Supplementary material for: Understanding Altered Dynamics in Cocaine Use Disorder Through State Transitions Mediated by Artificial Perturbations
Source: Brain Sci. 2025 Feb 28;15(3):263. doi: 10.3390/brainsci15030263 (PMC11939957; doi:10.3390/brainsci15030263)
Supplement: Supplementary file 1 [file brainsci-15-00263-s001.zip › brainsci-3478164-supplementary.pdf]

Supplementary Materials

Supplementary figures

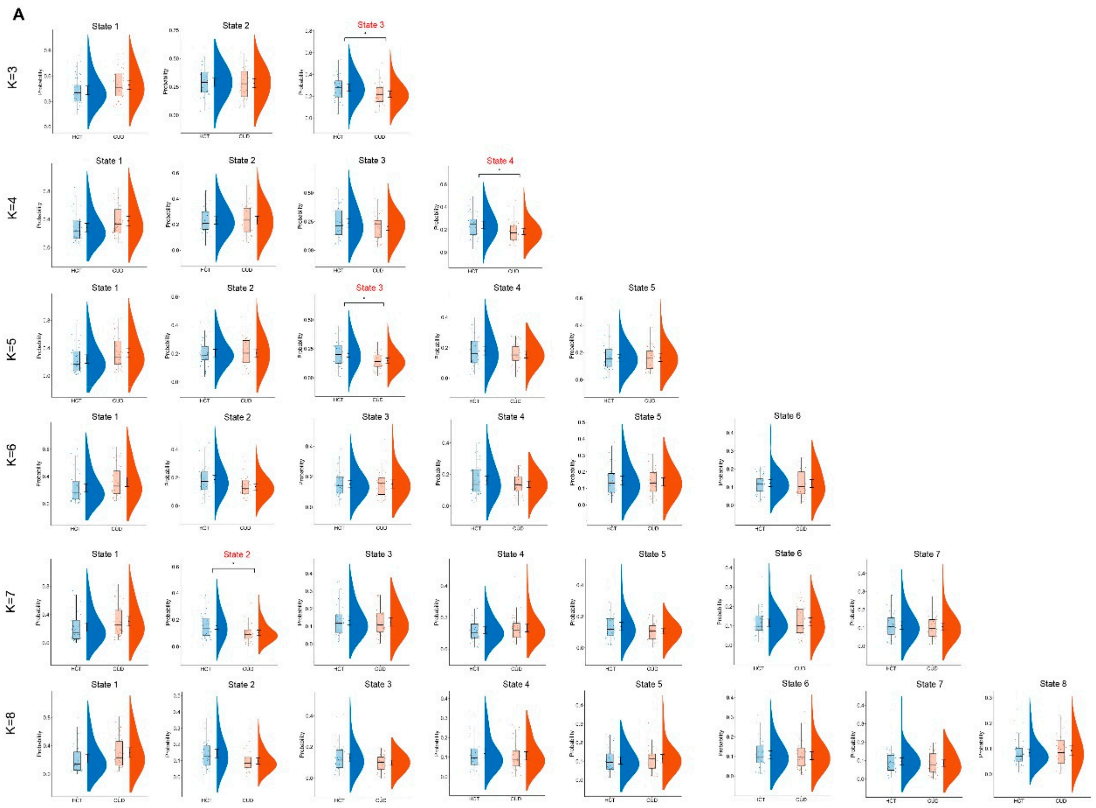

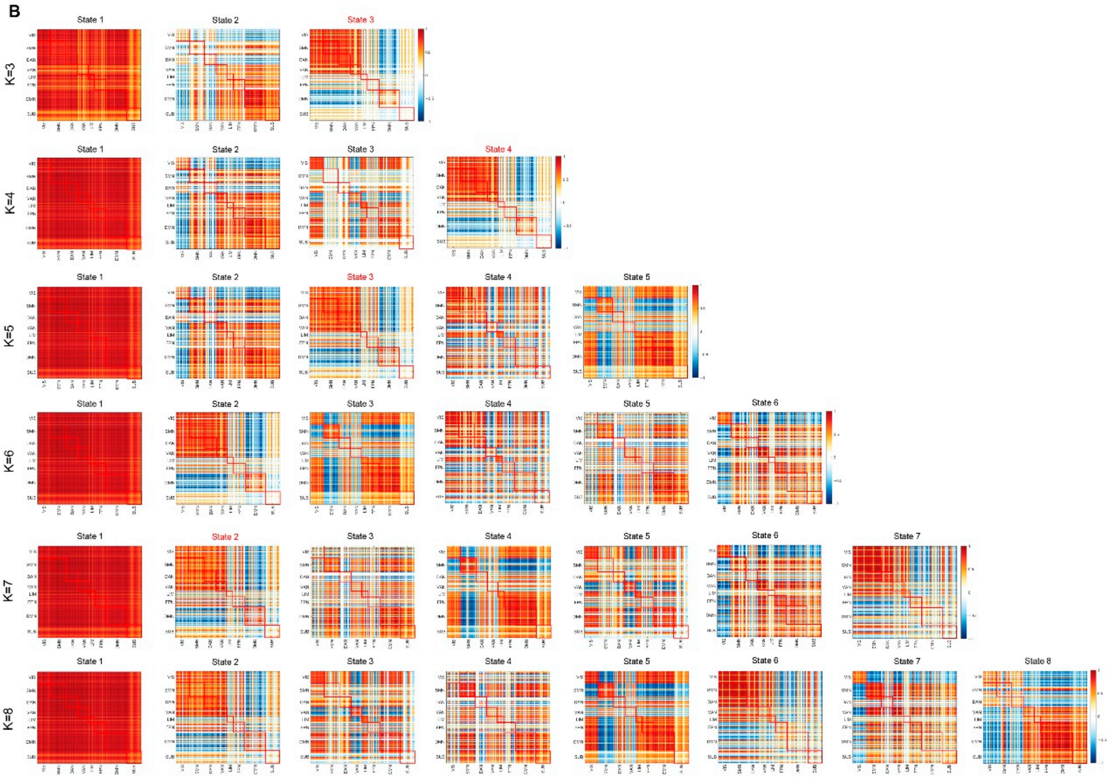

Figure S1. Clustering results for K=3-8 states. (A) Raincloud plots showing state occurrence probabilities across cluster number K. Violins (right portions of subplots) represent data distributions. Nodes and error bars next to violins represent mean values and 95% confidence intervals. Box plots (left portions of subplots) show the median (middle lines), interquartile ranges (box length) and 1.5 * quartile ranges (whiskers). Individual data points are jittered. The multivariate ANOVA tests controlling age, sex, education and mean framewise displacement showed significant differences in state probability distributions between HCT and CUD groups at $K = 3$ ($F(2,81) = 3.67$, $p < 0.05$), $K = 4$ ($F(3,80) = 3.41$, $p < 0.05$), $K = 5$ ($F(4,79) = 3.04$, $p < 0.05$), $K = 7$ ($F(6,77) = 2.37$, $p < 0.05$). Post-hoc analyses using univariate ANOVA showed significant differences in specific states ($K = 3$, state 3, $F(1,82) = 7.53$, corrected $p < 0.05$; $K = 4$, state 4, $F(1,82) = 8.80$, corrected $p < 0.05$; $K = 5$, state 3, $F(1,82) = 8.83$, corrected $p < 0.05$; $K = 7$, state 2, $F(1,82) = 8.83$, corrected $p < 0.05$). P-values were FDR-corrected using Benjamini-Hochberg procedure across states with * representing corrected $p < 0.05$, and the significant states are colored in red. (B) State-specific functional connectivity for each group. Regions are organized by the Yeo 7 networks. VIS: visual network, SMN: sensorimotor network, DAN: dorsal attention network, VAN: ventral attention network, FPN: frontoparietal network, DMN: default mode network.

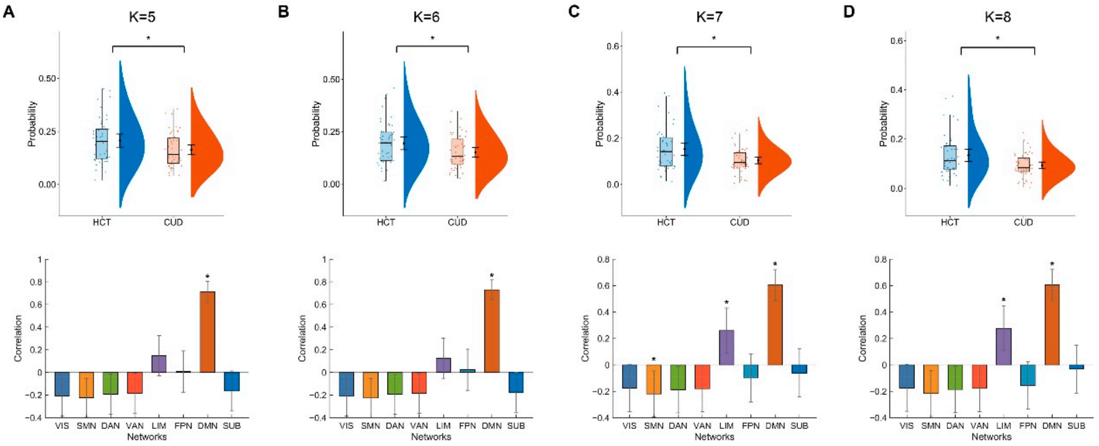

Figure S2. Robust analysis for the DMN-related state across different cluster number K. Probabilities of occurrence were computed and compared using CUD data from the SUDMEX TMS dataset. The upper panels are the raincloud plots of individual state probabilities and the statistical tests are made using univariate ANOVA controlling age, sex and mean framewise displacement. The lower panels are the Pearson correlations between states and Yeo 7 functional networks. (A) The state probability at K = 5 was significantly higher in the HCT condition than in the CUD condition (F(1,88) = 5.00, p < 0.05）. It positively correlated with DMN (r = 0.71, 95% CI [0.62, 0.80], FDR-corrected p < 0.005). (B) The state probability at K = 6 was significantly higher in the HCT condition than in the CUD condition (F(1,88) = 4.97, p < 0.05). It positively correlated with DMN (r = 0.73, 95% CI [0.64, 0.82], FDR-corrected p < 0.005). (C) The state probability at K = 7 was significantly higher in the HCT condition than in the CUD condition (F(1,88) = 9.74, p < 0.005). It positively correlated with DMN (r = 0.60, 95% CI [0.48, 0.72], FDR-corrected p < 0.005) and LIM (r = 0.26, 95% CI [0.09, 0.43], FDR-corrected p < 0.05) and negatively correlated with SMN (r = −0.22, 95% CI [−0.39, −0.05], FDR-corrected p < 0.05). (D) The state probability at K = 8 was significantly higher in the HCT condition than in the CUD condition (F(1,88) = 8.50, p < 0.005). It positively correlated with DMN (r = 0.61, 95% CI [0.49, 0.73], FDR-corrected p < 0.05) and LIM (r = 0.28, 95% CI [0.11, 0.45], FDR-corrected p < 0.05).

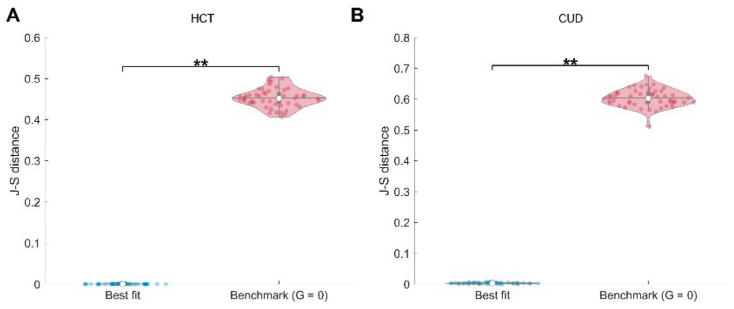

Figure S3. Comparisons of J-S distance between best fits and the benchmark (G = 0) using two-sample t-test. Data points represent 50 realizations of model optimization at fixed G. (A) Best fits of the HCT group showed significantly lower J-S distance than the benchmark (t(98) = −136, p < 0.001). (B) Best fits of the CUD group showed significantly lower J-S distance than the benchmark (t(98) = −149, p < 0.001). ** represents p < 0.001.

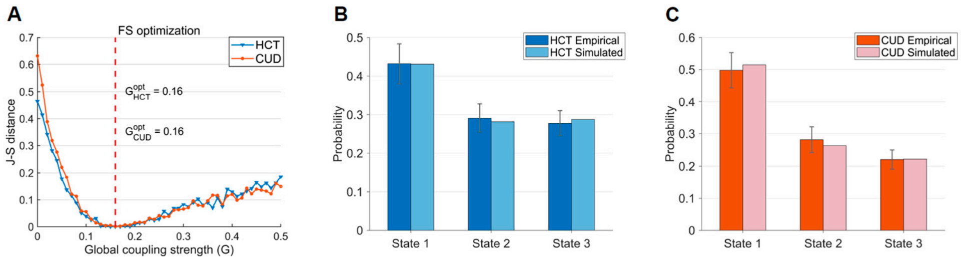

Figure S4. Whole-brain model performance with FS optimization alone. (A) J-S distance between empirical and simulated brain states as a function of global coupling strength G. The optimal fits were found at G = 0.16 (HCT and CUD). At each G value, the whole-brain model was optimized using FS. (B) The occurrence probabilities of empirical states and the corresponding best-fit states for HCT. (C) The occurrence probabilities of empirical states and the corresponding best-fit states for CUD. Error bars represent 95% confidence intervals. Bar plots summarize the group-level empirical and model performance metrics. Light-colored data represent simulated state probability dynamics aggregated over time intervals equivalent to scan durations of all individuals.

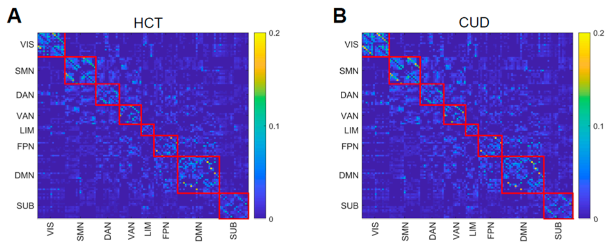

Figure S5. Effective connectivity (EC) generated by the FS and $\mathrm{FS}_{\mathrm{diff}}(\tau)$ optimization. (A) EC of the HCT model at its optimal fit. (B) EC of the CUD model at its optimal fit.

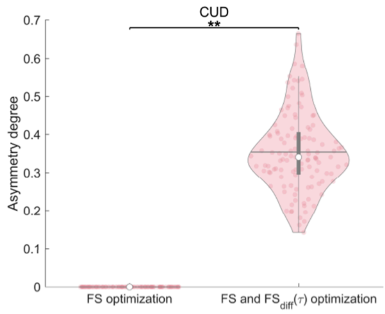

Figure S6. Violin plots of asymmetry degree under two optimization approaches for CUD. FS and $\mathrm{FS}_{\mathrm{diff}}(\tau)$ optimization induced stronger asymmetry of effective connectivity in the CUD condition ($t(115) = -37.0$, $p < 0.001$).

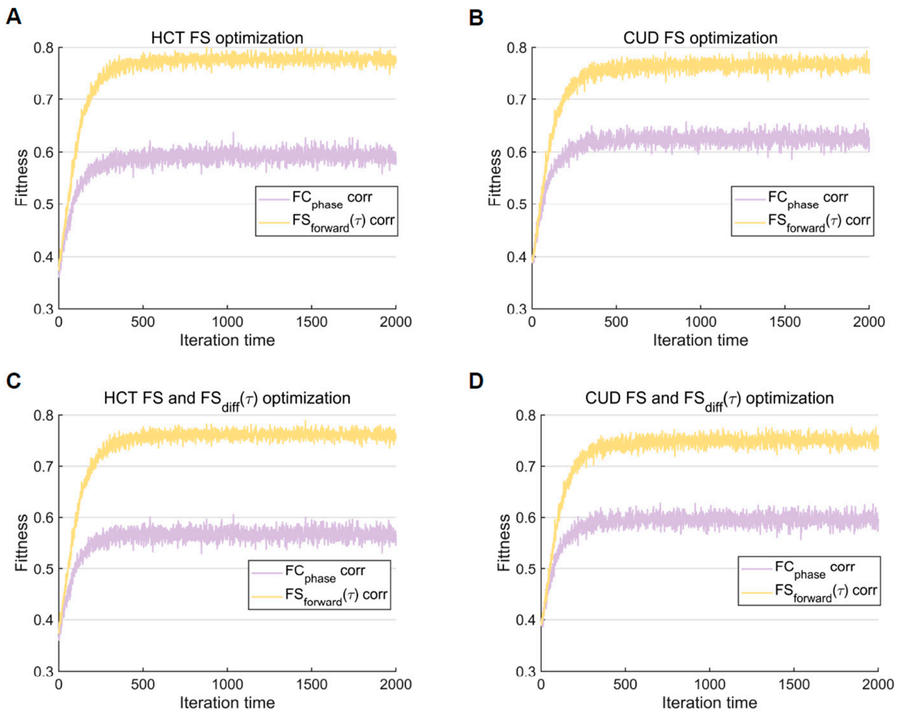

Figure S7. The evolution of correlations between empirical and simulated values of $\mathrm{FC}_{\mathrm{phase}}$ (yellow) and $\mathrm{FS}_{\mathrm{forward}}(\tau)$ (purple). (A) HCT model with FS optimization. (B) CUD model with FS optimization. (C) HCT model with FS and $\mathrm{FS}_{\mathrm{diff}}(\tau)$ optimization. (D) CUD model with FS and $\mathrm{FS}_{\mathrm{diff}}(\tau)$ optimization. Optimizing only FS also led to $\mathrm{FC}_{\mathrm{phase}}$ and $\mathrm{FS}_{\mathrm{forward}}(\tau)$ optimization.

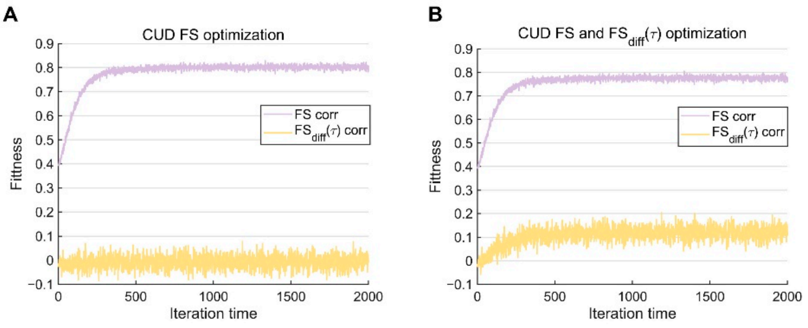

Figure S8. The evolution of correlations between empirical and simulated values of FS (purple) and $\mathrm{FS}_{\mathrm{diff}}(\tau)$ (yellow) for the CUD model. (A) FS optimization. (B) FS and $\mathrm{FS}_{\mathrm{diff}}(\tau)$ optimization.

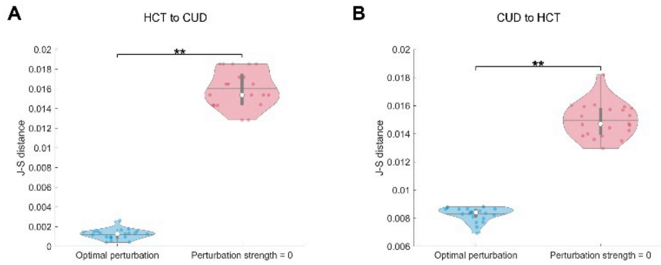

Figure S9. Comparisons of J-S distance between optimal perturbation configurations (n = 21) and the benchmark results with the same number (perturbation strength = 0) using two-sample t-test. (A) Optimal perturbations significantly drove the system from HCT toward CUD condition ($t(40) = -35.3$, $p < 0.001$). (B) Optimal perturbations significantly drove the system from CUD toward HCT condition ($t(40) = -23.3$, $p < 0.001$). ** represents $p < 0.001$.

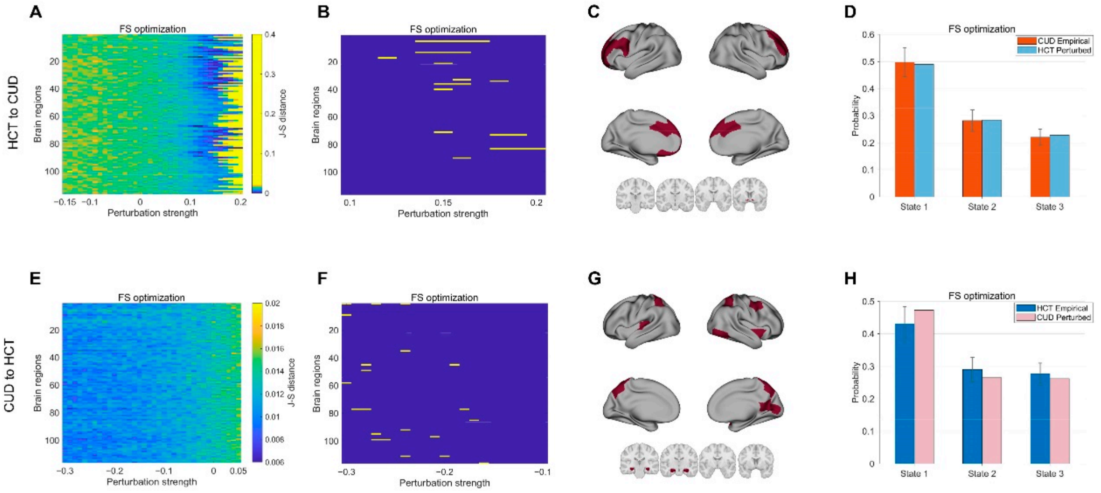

Figure S10. Perturbation results using FS optimization. (A) J-S distances between the empirical CUD states and perturbed HCT states across different strengths (−0.15 to 0.2) and regions. (B) Regions most sensitive to perturbations in (A) were identified by selecting the top 0.5% areas with minimal J-S distances. (C) Visualization of critical brain regions in (B). (D) Comparison of the state induced by stimulating the right dorsal prefrontal cortex and the empirical CUD state probabilities. (E) J-S distances between the empirical HCT states and perturbed CUD states across different strengths (−0.3 to 0.05) and regions. (F) Regions most sensitive to perturbations in (E) were identified by selecting the top 0.5% areas with minimal J-S distances. (G) Visualization of critical brain regions in (F). (H) Comparison of the state induced by stimulating the right retrosplenial cortex and the empirical HCT state probabilities. Bar plots summarize the group-level empirical and model performance metrics. Light-colored data represent model-stimulated state probability dynamics aggregated over time intervals equivalent to scan durations of all individuals.

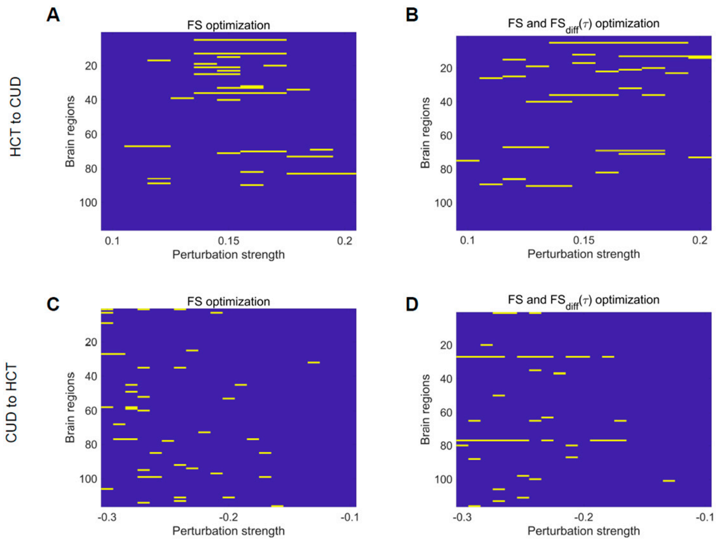

Figure S11. Sensitive brain regions selected using threshold 1% under different transitions and optimization methods. (A) Transition from HCT to CUD with FS optimization. (B) Transition from HCT to CUD with FS and $\mathrm{FS}_{\mathrm{diff}}(\tau)$ optimization. (C) Transition from CUD to HCT with FS optimization. (D) Transition from CUD to HCT with FS and $\mathrm{FS}_{\mathrm{diff}}(\tau)$ optimization.

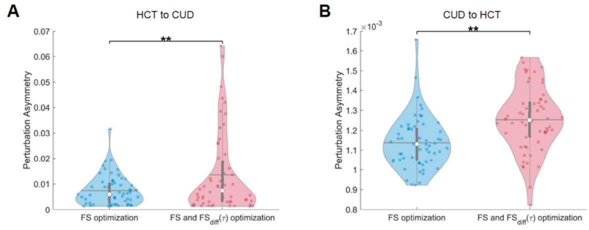

Figure S12. Perturbation asymmetry of state transitions. (A) Violin plots of perturbation asymmetry under two optimization approaches for transitions from HCT to CUD ($t(57) = 3.96$, $p<0.001$). (B) Violin plots of perturbation asymmetry under two optimization approaches for transitions from CUD to HCT (($t(57) = 3.51$, $p<0.001$)). The combined FS and $\mathrm{FS}_{\mathrm{diff}}(\tau)$ optimization significantly increased the perturbation asymmetry for both transitions. ** represents $p < 0.001$.

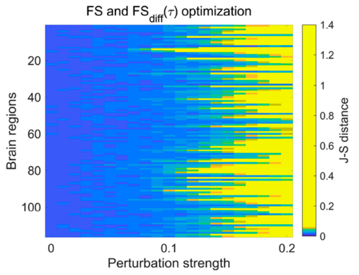

Figure S13. Perturbation results using FS and $\mathrm{FS}_{\mathrm{diff}}(\tau)$ optimization. J-S distances between the empirical HCT states and perturbed CUD states across different strengths (0 to 0.2) and regions.
